# β-cell insulin receptor deficiency during *in utero* development induces an islet compensatory overgrowth response

**DOI:** 10.18632/oncotarget.10342

**Published:** 2016-06-30

**Authors:** Mark Trinder, Liangyi Zhou, Amanda Oakie, Matthew Riopel, Rennian Wang

**Affiliations:** ^1^ Children's Health Research Institute, London, Ontario, Canada; ^2^ Departments of Physiology & Pharmacology, University of Western Ontario, London, Ontario, Canada; ^3^ Department of Pathology, University of Western Ontario, London, Ontario, Canada; ^4^ Department of Medicine, University of Western Ontario, London, Ontario, Canada

**Keywords:** inducible MIP-βIRKO, fetal pancreas, β-cell proliferation, islet vasculature, Pathology Section

## Abstract

The presence of insulin receptor (IR) on β-cells suggests that insulin has an autocrine/paracrine role in the regulation of β-cell function. It has previously been reported that the β-cell specific loss of *IR* (βIRKO) leads to the development of impaired glycemic regulation and β-cell death in mice. However, temporally controlled βIRKO induced during the distinct transitions of fetal pancreas development has yet to be investigated. We hypothesized that the presence of IR on β-cells during the 2^nd^ transition phase of the fetal murine pancreas is required for maintaining normal islet development. We utilized a *mouse insulin 1* promoter driven tamoxifen-inducible Cre-recombinase *IR* knockout (MIP-βIRKO) mouse model to investigate the loss of β-cell IR during pancreatic development at embryonic day (e) 13, a phase of endocrine proliferation and β-cell fate determination. Fetal pancreata examined at e19-20 showed significantly reduced IR levels in the β-cells of MIP-βIRKO mice. Morphologically, MIP-βIRKO pancreata exhibited significantly enlarged islet size with increased β-cell area and proliferation. MIP-βIRKO pancreata also displayed significantly increased Igf-2 protein level and Akt activity with a reduction in phospho-p53 when compared to control littermates. Islet vascular formation and Vegf-a protein level was significantly increased in MIP-βIRKO pancreata. Our results demonstrate a developmental role for the β-cell IR, whereby its loss leads to an islet compensatory overgrowth, and contributes further information towards elucidating the temporally sensitive signaling during β-cell commitment.

## INTRODUCTION

The insulin receptor (IR) is a receptor tyrosine kinase with widespread expression that promotes glucose uptake to peripheral tissue in response to secreted insulin from the pancreatic β-cells [1]. IR signaling is also implicated in cellular growth, differentiation, survival, and metabolism [2–4], and expression of IR in β-cells is critical for the maintenance of euglycemia and islet survival [5]. In patients with type 2 diabetes mellitus, a disease characterized by hyperglycemia due to insulin insensitivity or insufficient insulin secretion, the levels of IR and associated downstream signaling pathways are perturbed [6]. *In vitro* experiments with the mouse insulinoma (MIN6) cell line support the importance of IR function in β-cell physiology, where knockdown of *IR* expression resulted in dysregulation of over 1500 genes [7]. Notable cellular functions regulated by autocrine/paracrine β-cell insulin signaling include insulin production and glucose-stimulated insulin secretion [8]. β-cell IR signaling is required *in vivo* for adaptive islet hyperplasia in response to pancreatic injury and insulin resistance [9], and can enhance rescue from a hyperglycemic state, as seen in diabetic rats transplanted with rat insulinoma (INS-1) cells overexpressing human *IR* [10]. Alternatively, adult mice with β-cell specific *IR* knockout (βIRKO) displayed progressive impairments in glucose-stimulated insulin secretion, glucose tolerance, and maintenance of β-cell mass [11]. Although βIRKO mice have been reported to appear phenotypically normal at birth [9, 11], fetal and newborn mice with insulin deficiency exhibited increased islet size and proliferation associated with increased pancreatic capillary density and decreased islet apoptosis [12]. Taken together, insulin/IR signaling in β-cells could be an important regulator of islet development prenatally and critical for postnatal maintenance of β-cell function. Therefore, further investigation of IR activity in β-cells is essential to better understand the pathogenesis of diabetes and areas of interest for potential therapeutics.

Pancreatic development requires temporal regulation of both transcription factor expression and external signaling pathways to generate physiologically functional adult islets [13, 14]. In particular, the structural homology between the IR and insulin-like growth factor 1 receptor (Igf-1r) enables compensation between the two receptors, allowing β-cells to utilize overlapping signaling pathways to mediate similar functions [15]. This is particularly prominent during fetal development [16]. However, the temporal importance of IR signaling on the prenatal levels of transcription factors and metabolic proteins necessary for islet, and more specifically, β-cell differentiation, proliferation, maturation, and survival has yet to be sufficiently investigated. Thus, we proposed to determine if β-cell IR is an essential regulator of β-cell development to reconcile conflicting findings from the aforementioned adult βIRKO [11] and fetal insulin [12] knockout studies, and investigate potential adaptive signaling from the homologous Igf-1r. This is the first study to investigate the temporal role of the β-cell IR on the 2^nd^ transition period of fetal β-cell development by utilizing the *mouse insulin 1* promoter to drive tamoxifen-inducible Cre-recombinase (*MIP-CreER*) expression in the β-cell *IR* knockout (MIP-βIRKO) mouse model. Our results demonstrate that β-cell specific loss of IR during fetal β-cell development results in islet overgrowth due to significantly elevated levels of Igf-2, phospho-Akt and Vegf-a signals with associated β-cell replication.

## RESULTS

### Characterization of MIP-βIRKO knockout mice

**Figure 1:**
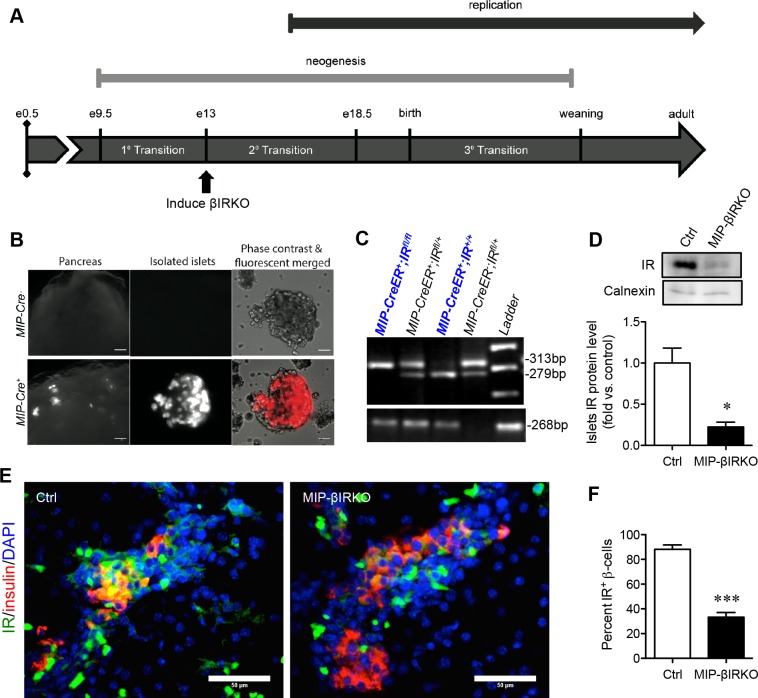
Confirmation of the fetal MIP-βIRKO mouse model **A.** Experimental model schematic with reference timeline of important events during islet development. **B.** Immunofluorescence image of Cre recombinase expression within pancreatic β-cells of *MIP-Cre*^+^, but not *MIP-Cre*^−^, mouse islets following cross-breeding to a B6.Cg-*Gt(ROSA)26Sor^tm9(CAG-tdTomato)Hze^*/J reporter strain. Scale bars: 25 μm (pancreas) and 5 μm (islets). **C.** Genotypes of fetal mice were determined by PCR of the *IR* and *MIP-CreER* genes, followed by subsequent gel electrophoresis. **D.** Western blotting demonstrated a significant reduction in insulin receptor (IR) protein level of fetal MIP-βIRKO pancreata relative to controls (*n* = 3-4). IR protein level was normalized to calnexin and expressed as fold *vs*. controls. Representative blotting is shown. **E.** Representative double immunofluorescence images and quantification of IR^+^ β-cells in MIP-βIRKO relative to control pancreatic sections. Scale bar: 50 μm. White bar, control group; black bar, MIP-βIRKO group. Data are expressed as means ± SEM. *p < 0.05, ***p < 0.001 *vs*. controls. e = embryonic day.

We investigated the temporal role of β-cell IR knockout on the 2^nd^ transition period of fetal β-cell development (Figure 1A), a crucial stage of pancreatic development characterized by endocrine cell proliferation and fate determination. To confirm that the IR knockout was specific to pancreatic β-cells, *MIP-Cre^+^* mice were first crossed with a B6.Cg-*Gt(ROSA)26Sor^tm9(CAG-tdTomato)Hze^*/J reporter strain. Fluorescence from the tdTomato reporter was specific to pancreatic β-cells (Figure 1B) and was not detected in the brain and other tissues, similar to results seen in other recent reports [17, 18]. The genotypes of mice were determined using PCR products of both the *IR* and *MIP-CreER* genes (Figure 1C, Supplemental Table 1). Experimental MIP-βIRKO (*MIP-CreER^+^*;*IR^fl/fl^*) mice were positive for both 313bp *IR^fl/fl^* and 268bp *MIP-CreER^+^* (Figure 1C); heterozygous *IR* (*IR^fl/+^*, 313bp and 279bp) (*MIP-CreER^+^*;*IR^fl/+^*) mice were excluded. Knockout of IR in β-cells was confirmed by western blot analyses and depicted a significant reduction in IR protein level (~75% lost) in MIP-βIRKO pancreata (*p* < 0.05 *vs*. controls; Figure 1D). Reduced IR staining in insulin^+^ β-cells of MIP-βIRKO mouse islets was also evaluated by double immunofluorescence staining (*p* < 0.001 *vs*. control, Figure 1E).

### Fetal MIP-βIRKO pancreas display an islet hyperplastic growth response

**Figure 2:**
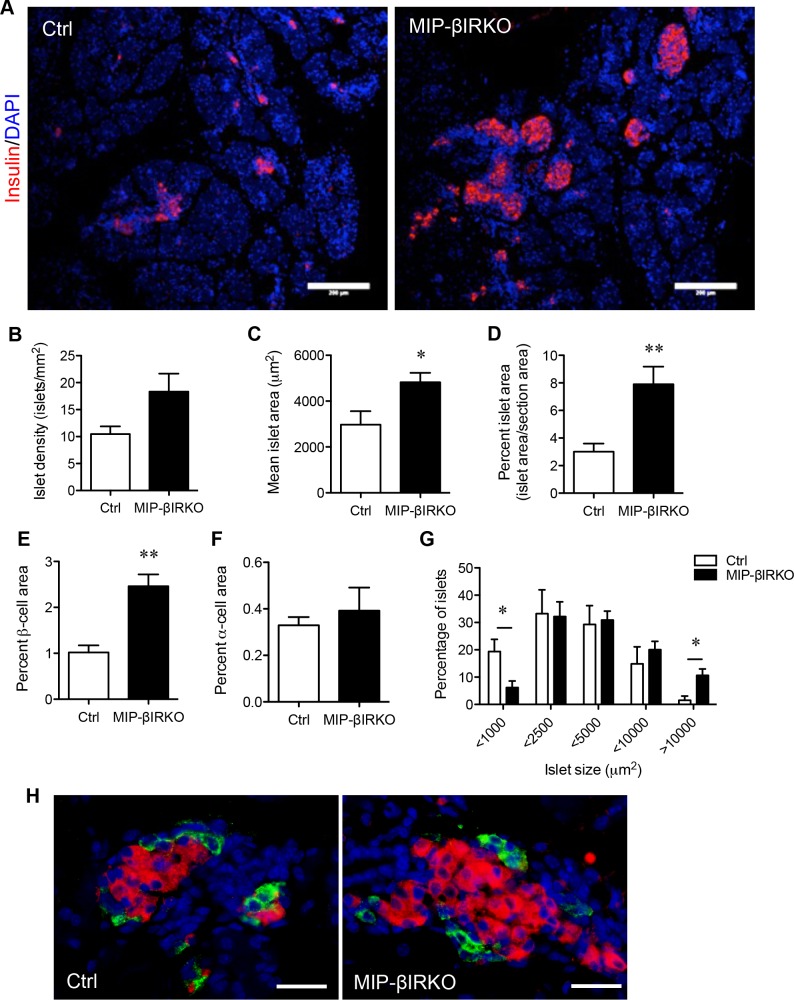
Fetal MIP-βIRKO mice display an islet hyperplastic growth response **A.** Representative immunofluorescence image depicting the expanded population of β-cells (red) within fetal MIP-βIRKO pancreata compared to controls. Scale bar: 200 μm. Morphometric analysis of **B.** islet density (islets/mm^2^), **C.** mean islet area, **D.** percent islet area, **E.** percent β-cell area, and **F.** percent α-cell area relative to whole pancreatic section area. **G.** Quantification of different islet sizes. White bars, control group; black bars, MIP-βIRKO group. Data are expressed as means ± SEM (*n* = 5-6). *p < 0.05, **p < 0.01 *vs*. controls. **H.** Representative double immunofluorescence images for glucagon (green) and insulin (red) staining in the islets of control and MIP-βIRKO mice. Scale bar: 50 μm. Nuclei were labeled with DAPI (blue).

Body weight and blood glucose for each control and MIP-βIRKO fetus were determined at the time of dissection (e19-20), with results indicating a similar general phenotype between the two studied groups (Supplementary Figure 1). In addition, there was no significant difference in circulating plasma insulin levels or insulin content from pancreatic tissue between control and MIP-βIRKO fetal mice (Supplementary Figure 2). Immunofluorescence staining of fetal MIP-βIRKO pancreatic sections revealed a higher total number of enlarged islets per pancreatic section (Figure 2A), and morphometric analyses revealed a 1.8-fold increase in islet density (number of islets per mm^2^ of pancreatic sections) in fetal MIP-βIRKO pancreata (Figure 2B). The mean islet area (μm^2^) (*p* < 0.05; Figure 2C), percent islet area (percent total islet area over total pancreatic section area; *p* < 0.01; Figure 2D), and percent β-cell area (percent total insulin^+^ area over total pancreatic section area; *p* < 0.001; Figure 2E) were significantly increased in the fetal MIP-βIRKO pancreas when compared to control pancreata. In contrast, α-cell area (percent total glucagon^+^ area to total pancreatic section area) did not differ between MIP-βIRKO and control groups (Figure 2F) with no alteration of α- and β-cell distributions (Figure 2H), suggesting that the β-cell specific *IR* knockout does not affect α-cells and islet structure. Furthermore, no changes were noted regarding somatostatin^+^- and pancreatic polypeptide^+^-cell populations in the islets (Supplementary Figure 3).

To determine if islet changes in MIP-βIRKO pancreata were due to increased islet number or due to larger islet sizes, we tallied the size distribution of islets in both controls and MIP-βIRKO islets according to a previously established method [12]. We observed that MIP-βIRKO pancreata had a significantly decreased small islet population (< 1000 μm^2^) and increased percentage of large islets (> 10000 μm^2^) compared to controls (Figure 2G).

### Fetal MIP-βIRKO increase β-cell proliferation with no change in apoptosis

**Figure 3:**
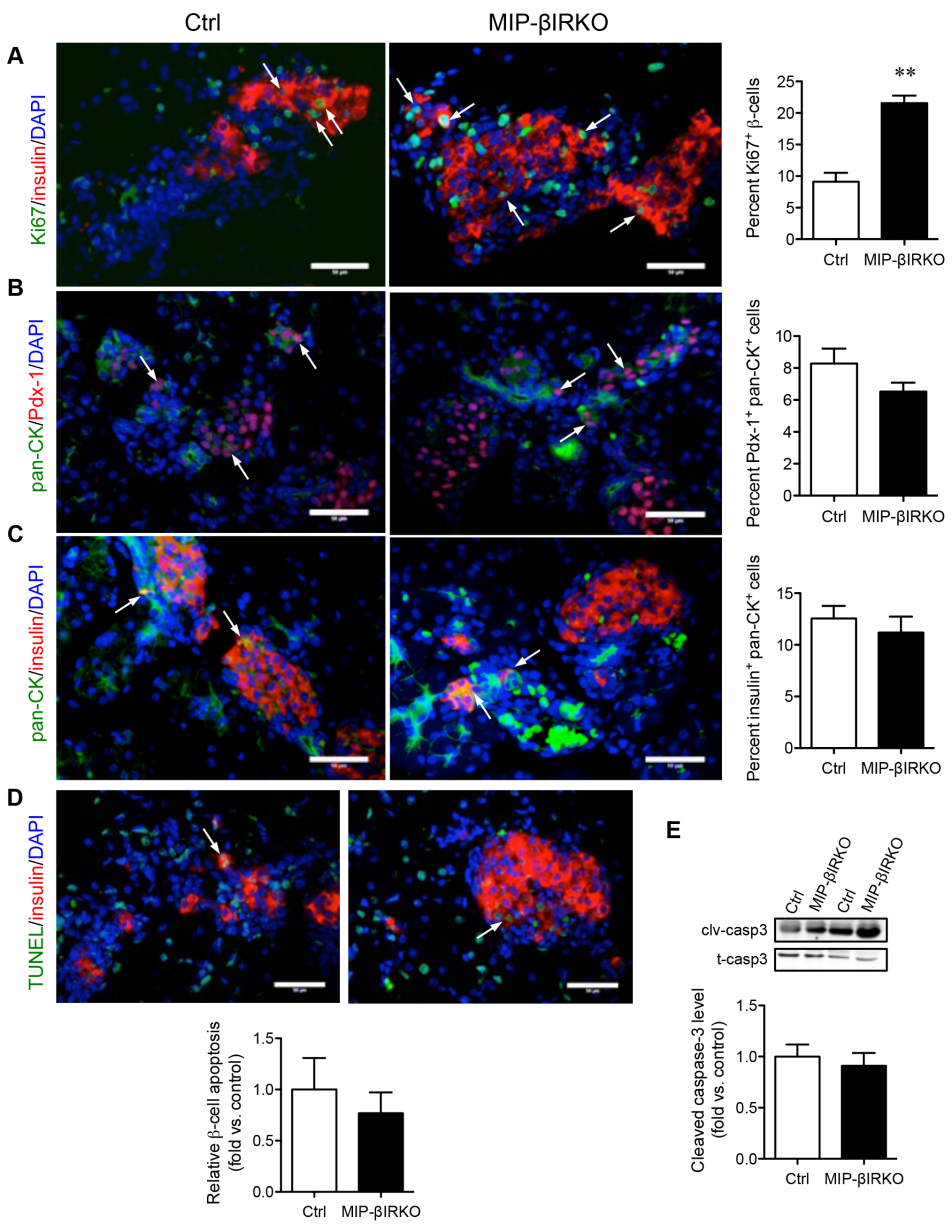
Fetal MIP-βIRKO mice show increased β-cell proliferation with no change in apoptosis Representative double immunofluorescence images: **A.** Ki67/insulin, **B.** pan-CK/Pdx-1, **C.** pan-CK/insulin, and **D.** TUNEL/insulin of MIP-βIRKO and control pancreatic sections. Nuclei stain, DAPI (blue). White arrows indicate double positive cells. Scale bar: 50 μm. Quantification of Ki67^+^ β-cells, Pdx1^+^/Pan-CK^+^ cells, insulin^+^/Pan-CK^+^ cells and β-cell apoptosis in MIP-βIRKO islets relative to controls. **E.** Western blot analysis of cleaved caspase-3 (clv-casp 3) and total caspase-3 (t-casp 3). Data is normalized to T-Casp 3 and expressed as fold change to controls. White bars, control group; black bars, MIP-βIRKO group. Data are expressed as means ± SEM (*n* = 4-5). ***p < 0.01 *vs*. controls.

To deduce a potential explanation for the islet growth response, we evaluated β-cell proliferation, death, and neogenesis in MIP-βIRKO mice. Double immunofluorescence staining for insulin and Ki67 was used to quantify the proliferating insulin^+^ cells. Staining showed that Ki67^+^/insulin^+^ co-localization was significantly higher (2.4-fold) in MIP-βIRKO pancreata compared to controls (*p* < 0.01, Figure 3A). During both fetal and postnatal development, islet neogenesis is initiated from the pancreatic ductal epithelium [19, 20]. As an indicator for islet neogenesis, we evaluated the number of ductal cells displaying early commitment to the β-cell lineage by dual labeling of Pan-CK with Pdx-1 (marker for pancreatic progenitors) or insulin (Figure 3B, 3C). The percentage of Pan-CK^+^ cells expressing nuclear Pdx-1 showed no significant difference between MIP-βIRKO (7%) and control (8%) pancreata (Figure 3B). A similar result was observed for the percentage of insulin^+^ ductal cells (Figure 3C). These findings suggest that the islet growth seen in MIP-βIRKO mice is likely attributed to the increased replication of pre-existing β-cells, rather than ductal-to-islet neogenesis.

To assess the possibility that decreased apoptosis of developing islet cells could also contribute to the islet compensatory response seen in MIP-βIRKO mice, a TUNEL assay was performed. The percentage of insulin^+^ cells labeled by TUNEL in MIP-βIRKO and control pancreas was similar (Figure 3D). Western blot analyses also revealed no difference in caspase-3 cleavage between MIP-βIRKO and control pancreata (Figure 3E). This data indicates that the enriched β-cell population of the MIP-βIRKO pancreas is not due to decreased β-cell apoptosis.

### Fetal MIP-βIRKO islets show enhanced replication and pro-survival signaling pathway activity

**Figure 4:**
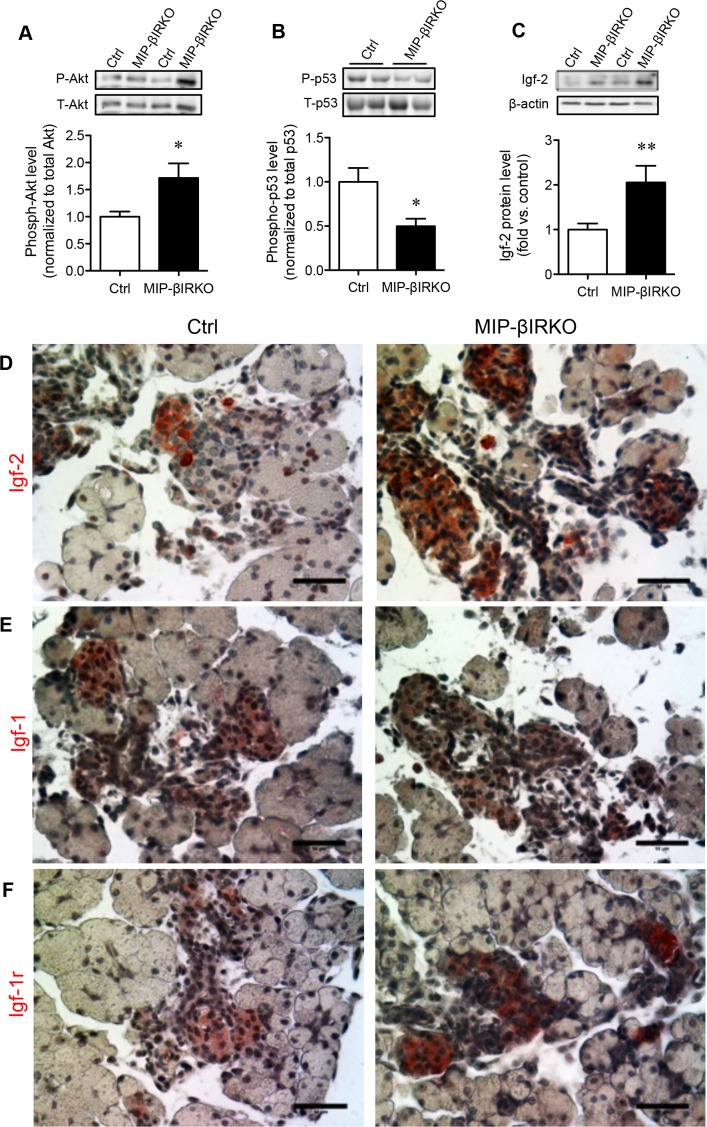
Fetal MIP-βIRKO islets have enhanced replication and pro-survival signaling pathway activity Western blot analyses of phospho-S473 Akt (P-Akt) and total Akt (T-Akt) (**A.**, *n* = 4), phospho-S15 p53 (P-p53) and total p53 (T-p53) (**B.**, *n* = 3-4), and Igf-2 protein level (**C.**, *n* = 5-13) in fetal pancreata from MIP-βIRKO and control mice. Representative blotting images are shown. White bar, control group; black bar, MIP-βIRKO group. Data are expressed as means ± SEM. *p < 0.05, **p < 0.01 *vs*. controls. Representative immunohistochemical images for **D.** Igf-2, **E.** Igf-1, and **F.** Igf-1r staining of MIP-βIRKO and control pancreatic sections. Nuclei were counterstained with hematoxylin. Scale bar: 50 μm.

We next investigated the possible mechanistic link between IR and Igf-1r signaling cascades relevant to enlarged islets in the fetal MIP-βIRKO pancreas. The PI3K/Akt signaling pathway plays a critical role in the regulation of β-cell replication [21]. We observed significantly increased phospho-Akt (S473) levels in fetal MIP-βIRKO pancreata compared to controls (*p* < 0.05; Figure 4A). In line with up-regulation of phospho-Akt, a significantly reduced level of phoshpo-p53 (S15), a marker for proapoptotic p53, was also observed in MIP-βIRKO pancreata relative to controls (*p* < 0.05; Figure 4B). To determine whether the developmental compensation for impaired β-cell IR signaling could be mediated by the action of the homologous Igf-1r signaling pathway, the levels of pancreatic Igf-1 and Igf-2 were examined. Igf-2 protein levels were significantly elevated in the pancreas of fetal MIP-βIRKO mice compared to controls (*p* < 0.01; Figure 4C), with an observable increase in Igf-2 staining intensity in MIP-βIRKO islets (Figure 4D). In contrast, both control and MIP-βIRKO fetal islets had low levels of Igf-1 (Figure 4E). Pancreatic immunohistochemistry also demonstrated stronger staining-intensity of Igf-1r in MIP-βIRKO islets (Figure 4F).

### Fetal MIP-βIRKO islets show increased Vegf-a levels with enriched islet-vasculature

**Figure 5:**
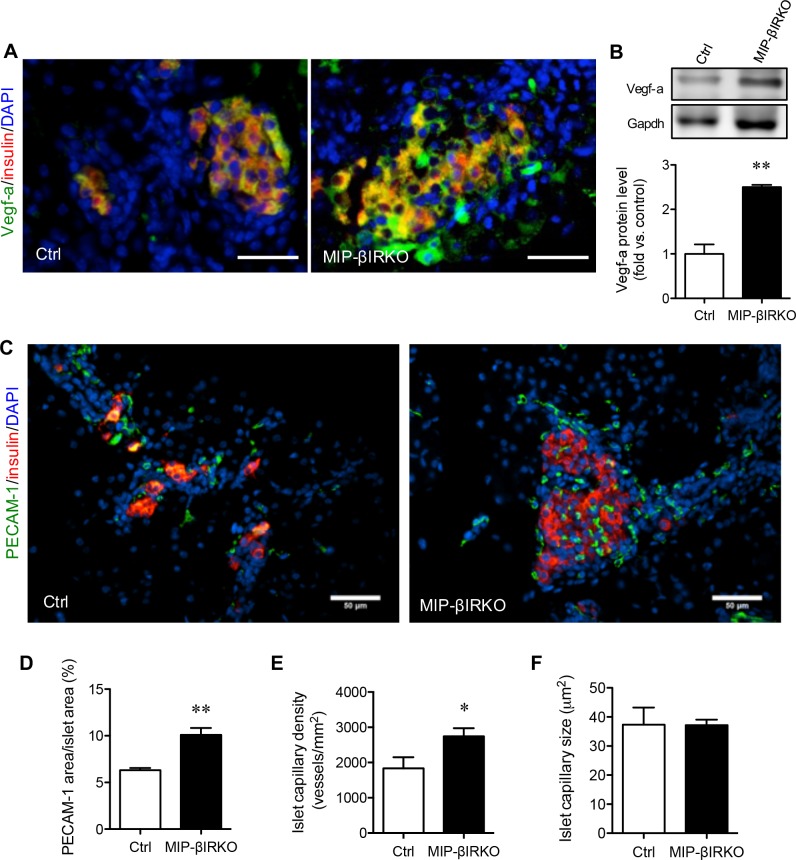
Fetal MIP-βIRKO islets show enhanced islet vascularization Representative double immunofluorescence images for **A.** Vegf-a or **C.** PECAM-1 (green) with insulin (red), and nuclei stain DAPI (blue), of MIP-βIRKO and control pancreatic sections. Scale bar: 50 μm. **B.** Western blotting analysis of Vegf-a protein level in fetal pancreas of MIP-βIRKO and control mice (*n* = 3-5). Representative blotting image is shown. Quantitative analyses of **D.** percent PECAM-1^+^ area in the islets, **E.** islet capillary density and **F.** average islet capillary size in MIP-βIRKO and controls pancreata. White bars, control group; black bars, MIP-βIRKO group. Data are expressed as means ± SEM (*n* = 4-5). *p < 0.05, **p < 0.01 *vs*. controls.

We further evaluated whether up-regulation of Igf-2 and Akt signaling could enhance islet Vegf-a levels, a major factor associated with increased islet growth [22], and islet vascularization in the developing fetal pancreas. We found that MIP-βIRKO islets had elevated pancreatic Vegf-a protein levels as determined by immunofluorescence (Figure 5A) and western blotting (*p* < 0.01 *vs*. control islets; Figure 5B). Morphological quantification of islet vascularization (Figure 5C) was performed by measuring the PECAM-1^+^ area, islet capillary density, and average capillary diameter. The percentage of islet PECAM-1^+^ area over islet area (*p* < 0.01; Figure 5D) and islet capillary density (number of islet capillary per islet area) (*p* < 0.05; Figure 5E) were significantly increased in MIP-βIRKO mice compared to controls. No change in the average islet capillary size between MIP-βIRKO and control groups was observed (Figure 5F). In accordance with these findings, MIP-βIRKO mice demonstrated increased islet angiogenesis, as opposed to enlargement of pre-existing capillaries.

### MIP-βIRKO at 2^nd^ developmental transition stage does not affect early β-cell identity

**Figure 6:**
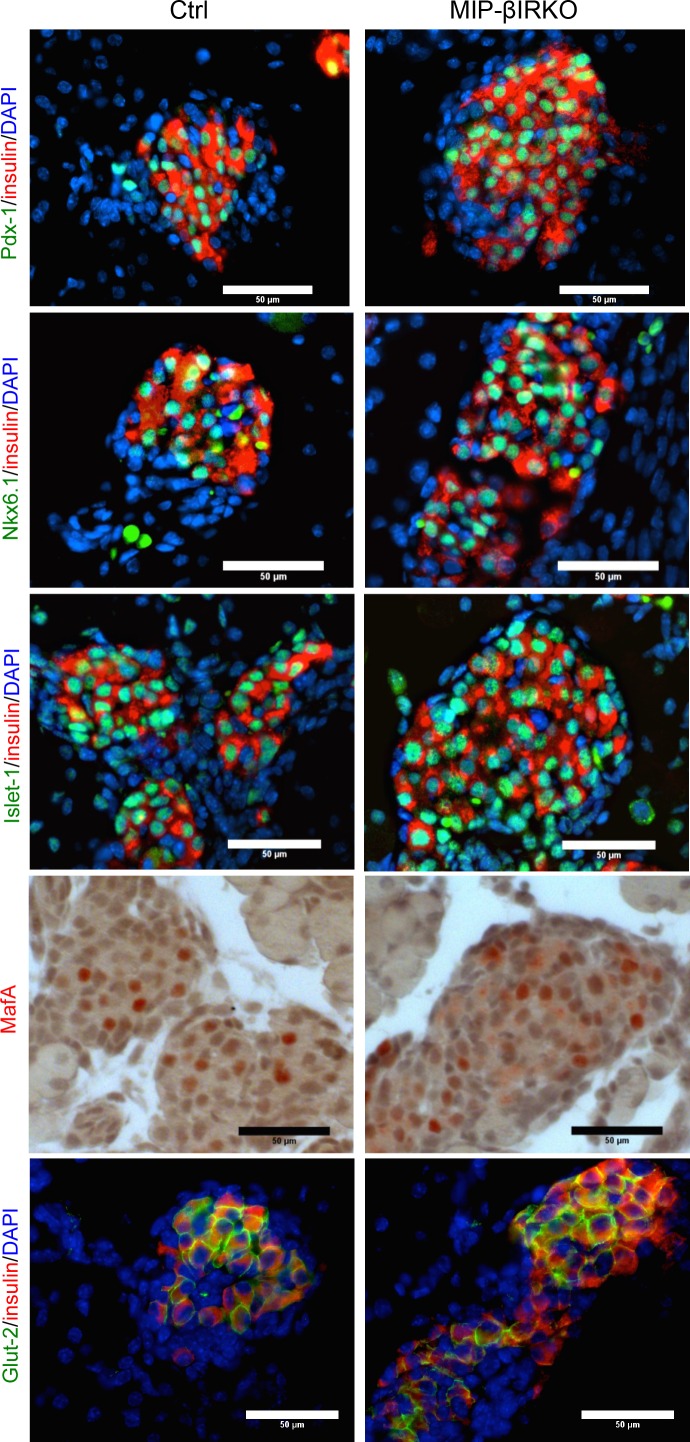
β-cells of fetal MIP-βIRKO islets display typical markers of β-cell identity and function Representative double immunofluorescence images for Pdx-1, Nkx6.1, Isl-1 and Glut-2 (green) with insulin (red), and nuclei stain DAPI (blue), of MIP-βIRKO and control pancreatic sections. Immunohistochemical staining for MafA (red in nuclei). Scale bar: 50 μm.

Previous studies have shown that impairments to the IR-signaling pathway result in reduced expression and nuclear-to-cytoplasmic translocation of important β-cell transcription factors, notably seen with factors Pdx-1 and MafA [7, 9]. Therefore, we employed double immunostaining on whole fetal pancreatic tissue sections to investigate potential dysregulation of transcription factors critical for development and maintenance of β-cell identity and function. Qualitative observation revealed that approximately all insulin^+^ cells possessed nuclear Pdx-1, Nkx6.1, Islet-1 and MafA (Figure 6) in both MIP-βIRKO and control groups. Despite the presence of larger islets in MIP-βIRKO mice, we observed similar intensity and membrane-localization of Glut-2 staining in the insulin^+^ cells of MIP-βIRKO and control islets (Figure 6). These results suggest that β-cell identity in MIP-βIRKO islets is maintained throughout fetal development and is independent from β-cell IR loss at the 2^nd^ transitional stage of islet development.

## DISCUSSION

**Figure 7:**
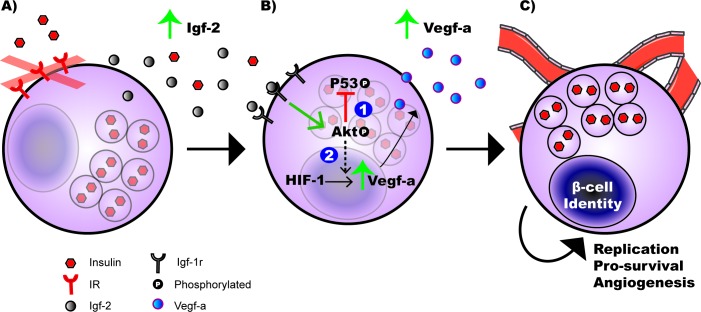
Working model of hyperplastic islet response observed in fetal MIP-βIRKO mice **A.** The loss of insulin receptor (IR) expression on pancreatic β-cells during 2^nd^ transition of the pancreatic development results in enhanced secretion of insulin growth factor-2 (Igf-2). **B.** Autocrine/paracrine activation of the insulin growth factor-1 receptor (Igf-1r) on pancreatic β-cell by Igf-2 promotes Akt phosphorylation, leading to: (1) suppressed phospho-p53 (S15) activation that prevents β-cell apoptosis, and (2) increased secretion of vascular endothelial growth factor-a (Vegf-a) through the Akt/HIF-1/Vegf-a signaling pathway in β-cells [22]. **C.** Together, Vegf-a-mediated hyper islet vascularization and upregulated Akt activity promotes exaggerated β-cell replication and cell survival, leading to an expanded β-cell population within the pancreas of MIP-βIRKO animals.

To evaluate the *in vivo* role of β-cell IR activity during the 2^nd^ transition phase of pancreatic endocrine development, we utilized a conditional and temporal MIP-βIRKO mouse model. We found that the fetal MIP-βIRKO mouse pancreas displays significantly increased mean islet and β-cell area, mainly attributed to enhanced pre-existing β-cell replication rather than islet neogenesis or apoptosis. The contribution of islet overgrowth phenomena is potentially associated with significantly enhanced Igf-2 production *via* Igf-1r in islets, with corresponding increased Akt phosphorylation, and enriched islet vasculature due to increased Vegf-a production (Figure 7). Our results indicate the abrupt interruption of typical autocrine/paracrine β-cell insulin signaling during the 2^nd^ transitional phase of pancreatic endocrine development leads to an islet compensatory overgrowth.

The transcription factors Pdx-1, Nkx6.1, Isl-1, and MafA are essential for early pancreatic endocrine development, maintenance of β-cell identity, and regulation of β-cell function in mature islets, while Glut2 is necessary for glucose uptake in β-cells and regulates the appropriate insulin release response [23–26]. Similar to previous studies that examined a non-inducible βIRKO mouse model [11], we did not observe any evidence for alteration of β-cell identity in fetal MIP-βIRKO pancreata as demonstrated by insulin^+^ cells co-localizing to nuclear Pdx-1, Nkx6.1, Isl-1, MafA and membrane Glut2. Although previous IRKO studies suggest that the β-cell IR plays an insignificant role in fetal islet development [9, 11], the discrepancy between results from other studies and the findings presented in our own may be due to the following: (1) knockout timing during development, (2) missing in-depth characterization of islet morphology in prior reports, and (3) the previous utilization of the alternative *rat insulin 2* promoter to drive Cre-recombinase expression specifically in β-cells [9, 11]. This is important because mice expressing Cre-recombinase *via* the *rat insulin 2* promoter show ectopic Cre-recombinase expression in the brain [27], which is a key region for endocrine pancreatic regulation [28]. High-resolution microscopy analysis of a reporter line has determined that the *MIP-CreER* mouse model used in this study does not drive the excision of floxed genes in the central nervous system [17, 29]. It is acknowledged that ectopic human growth hormone (hGH) was highly expressed in MIP-CreER islets independent of tamoxifen administration [17], which might promote the paracrine effects of hGH-induced serotonin expression [30]. To clarify this possible influence on our results, we examined the islet morphologies of MIP-CreER negative IR knockout (*MIP-CreER^−^;IR^fl/fl^*) and MIP-CreER positive wide-type (*MIP-CreER^+^*;*IR^+/+^*) mouse pancreas during *in utero* development and did not detect any differences between these two groups. Thus, the findings indicate no major effect of hGH at the fetal development stage.

Histological analyses of islets demonstrated that fetal MIP-βIRKO β-cells have significantly increased proliferative capacity *in vivo* over their control β-cell counterparts. Murine β-cell neogenesis begins at e9 and remains active for the first three weeks after birth [31], and by the end of the 3^rd^ transition stage is rapidly diminished and replaced by β-cell replication. We observed similar islet neogenesis from epithelial ducts in both MIP-βIRKO and control pancreata. Therefore, the islet compensatory response observed in MIP-βIRKO mice is likely a result of enhanced β-cell replication and expansion of individual islets as opposed to β-cell neogenesis. This is congruent with the observation of increased Akt activity, an important regulator of β-cell proliferation and anti-apoptotic signaling [21], in fetal MIP-βIRKO pancreata. The up-regulated activity of the PI3K/Akt pathway is potentially induced by elevated Igf-2 activation from compensatory Igf-1r signaling in the fetal MIP-βIRKO pancreas (Figure 7). Previous research suggests the IR and Igf-1r can compensate for one another since mice lacking both receptors specifically in β-cells died from diabetic ketoacidosis within 4-8 weeks [32]. *In vitro* β-cell IRKO experiments support this study's observations with compensatory increase in Igf-1r levels [33]. Similarly, an Igf-2 compensatory response to *in vivo* β-cell IR loss is logical since Igf-2 levels are higher than Igf-1 during fetal development and its expression is strongly localized to islets [16]. Igf-2 is important for promoting islet cell hyperplasia and apoptosis inhibition, which would account for the changes seen in fetal MIP-βIRKO pancreata. Our findings of reduced phospho-p53 (S15) in the MIP-βIRKO pancreas, relative to controls, corroborates with our observation of upregulated active Akt in MIP-βIRKO pancreas as Akt is often correlated with decreased downstream p53 activity (Figure 7). Since active p53 is critical for cell cycle arrest and DNA repair as well as apoptosis in response to DNA damage, we speculate that impaired regulation of cell cycle arrest and subsequent DNA repair could be important for increased β-cell proliferation seen in MIP-βIRKO pancreata [34, 35]. Furthermore, this mechanism could potentially account for the β-cell atrophy that has been observed in adult βIRKO mice [11].

Our findings are consistent with studies in congenital insulin knockout mice that show increased islet proliferation and islet vascularization during fetal pancreas development [12]. MIP-βIRKO pancreata demonstrated a significant increase in the percent of PECAM-1^+^ area and islet capillary density, and higher Vegf-a protein levels. This data matches our observation of enhanced Igf-2 levels in MIP-βIRKO pancreata, as vascular endothelial growth factor (Vegf) can be upregulated by *Igf-2* expression [36]. Igf-2 acts as a stimulatory ligand of both the Igf-1r and IR, and is therefore a critical factor for expansion of β-cell mass [37–39] and embryonic vasculogenesis by up-regulating Vegf levels *via* the Akt signaling pathway (Figure 7) [40]. Our recent study further determined that Vegf-a synthesis and secretion in the β-cell is regulated by the Akt/mTOR pathway, through increased *HIF-1* expression, and is associated with increased β-cell mass and proliferation [22]. Since the endothelium and its associated blood supply are critical for maintenance of β-cell fate and proliferation, numerous studies have proposed that increased islet vascularization could result in enhanced β-cell replication through rapid and efficient exchange of nutrients and hormones [12, 41–44]. In fact, short-term β-cell specific *Vegf-a* overexpression in mice resulted in pancreatic islet hypervascularization simultaneous with increased β-cell proliferation [44]. It is important to note that Igf-2 levels rapidly diminish after birth in rodents, thus further studies are needed to examine whether the increased islet growth seen in fetal MIP-βIRKO pancreas can be sustained in postnatal life and to determine whether these mice acquire postnatal glucose intolerance or develop innate protection against age-dependent islet mass degeneration [9, 11]. We utilized multiple protocols to produce and maintain viable neonatal offspring after intraperitoneal tamoxifen administration at e13 to pregnant mothers, but all attempts were largely unsuccessful due to pup rejection or non-viable progeny. Thus, a later time-point for the temporal induction of β-cell IRKO in pregnant mothers will be required.

In summary, this is the first study to temporally knock out the β-cell IR during the 2^nd^ transition phase of pancreatic endocrine development. Fetal MIP-βIRKO pancreata displayed an enlarged islet size and increased β-cell area that is likely associated with the observation of high β-cell proliferation in response to increased islet vascularization. Furthermore, we have demonstrated that fetal MIP-βIRKO is accompanied by heightened Igf-2/Igf-1r signaling, presumably leading to the activation of Akt and subsequent proliferation and enriched islet vasculature *via* increase of Vegf-a. Taken together, an understanding of the physiological function of insulin signaling on fetal β-cells is crucial for preventing abnormal islet development *in utero* and fine-tuning the niche for optimal *in vivo* survival of β-cells.

## MATERIALS AND METHODS

### Generation of inducible β-cell specific insulin receptor knockout mice (MIP-βIRKO)

*B6.129S4(FVB)-Insr^tm1Khn^/J* (*IR^fl/fl^*) and *Tg(Ins1-Cre/ERT)^1Lphi^* (*MIP-CreER*) were obtained from the Jackson Laboratories (Bar Harbor, MA, USA; stock number: 006955) and Dr. Louis Philipson's laboratory (University of Chicago, Chicago, IL, USA) [18], respectively. *MIP-CreER* and *IR^fl/fl^* mice were crossed at our facilities. The resulting *MIP-CreER:IR^fl/+^* mice were timely mated to generate experimental fetal groups. All mice were provided *ad libitum* access to both food and water. All animal use protocols were approved by the Animal Use Subcommittee at Western University in accordance with the Canadian Council of Animal Care.

Tamoxifen (Sigma; St. Louis, MO, USA) was prepared by dissolving in ethanol and suspending in corn oil (Sigma). A single dose of 6mg/40g body weight was administered by i.p. injection to pregnant mice at e13 (Figure 1A). At e19-20, pregnant mice were euthanized and immediately dissected to obtain the fetal tail for genotyping and pancreas for protein extraction and morphological analyses. Isolated fetuses were measured for body weight and blood glucose, and genotype was determined using PCR primers for the *Insr^tm1Khn^* mutation and the *MIP-CreER* promoter (listed in Supplementary Table 1). Experimental MIP-βIRKO (*MIP-CreER^+^*;*IR^fl/fl^*) mice were *MIP-CreER*^+^ (268bp) with both *IR* alleles floxed by *loxP* sites (*IR^fl/fl^*, 313bp). Control animals included *MIP-CreER^−^* with *IR* alleles floxed (*MIP-CreER^−^;IR^fl/fl^*) and *MIP-CreER^+^* with *IR* wild-type (*IR^+/+^*, 279bp) (*MIP-CreER^+^*;*IR^+/+^*). We confirmed that the phenotypes of *MIP-CreER^−^;IR^fl/fl^* and *MIP-CreER^+^*;*IR^+/+^* were virtually identical in terms of islet morphology measurements.

### ELISA assay for insulin

Fetal blood plasma and pancreata were collected at e19-20 from control and MIP-βIRKO mice. Blood was collected from fetal mice by decapitation and exsanguination immediately after the mother was sacrificed. Pancreatic insulin was extracted by acid-ethanol extraction buffer and homogenized. Both plasma and pancreatic insulin content were measured using an ultrasensitive (mouse) insulin ELISA kit (ALPCO, Salem, NH). Plasma insulin level was expressed as ng per ml, and pancreatic insulin content was normalized to protein and expressed as ng per mg protein [22].

### Tissue processing, immunostaining, and TUNEL

Dissected fetal pancreata were fixed in 4% paraformaldehyde and embedded in paraffin as previously described [22]. Sections (2-4μm thick) were prepared and stained with appropriately diluted primary antibodies (Supplementary Table 2), and detected using fluorescently-labeled secondary antibodies (Jackson Immunoresearch Laboratories; West Grove, PA, USA). Nuclei were counterstained with DAPI (Sigma-Aldrich; St. Louis, MO, USA). Islet Igf-1, Igf-1r, Igf-2 and V-maf avian musculoaponeurotic fibrosarcoma oncogene homolog A (MafA) levels were determined by immunohistochemical staining using the streptavidin-biotin horseradish peroxidase complex and developed with aminoethyl carbazole substrate kit (Invitrogen, Burlington, ON, Canada). Apoptotic β-cells (insulin^+^) were identified by the terminal deoxynucleotidyl transferase dUTP nick end-labeling (TUNEL) assay using an *In Situ* Cell Death Detection Kit (Roche Applied Science, Quebec City, QC, Canada).

Images of tissue sections were captured and islet morphologies were blindly analyzed with Image-Pro Plus software (Media Cybernetics; Rockville, MD, USA) for islet density, total islet area, islet sizes, and total α-cell or β-cell area. Islet vasculature including platelet endothelial cell adhesion molecule-1^+^ (PECAM-1) area per islet, islet capillary density and average islet capillary size were measured [22]. An islet was defined as a dense cell cluster containing at least 3 insulin^+^ cells and a glucagon^+^ cell [45]. Co-localization of Ki67, transcription factors, or Glut2 with insulin^+^ cells were also determined by double immunofluorescence or immunohistochemical staining [22].

### Protein extraction and western blot analyses

Fetal pancreas, muscle, liver, and brain tissues were sonicated in Nonidet-P40 lysis buffer. Protein lysates were separated by 5-15% SDS-PAGE and were wet transferred to a nitrocellulose membrane (Bio-Rad Laboratories; Mississauga, ON, Canada), then incubated with appropriately diluted primary (Supplementary Table 2) and secondary horseradish peroxidase-conjugated secondary antibodies. Proteins were visualized with Western Lightning Plus-Enhanced Chemoluminescence reagents (PerkinElmer; Waltham, MA, USA). All images were recorded using a Versadoc Imaging System (Bio-Rad Laboratories). Densitometric analyses of images were performed at subsaturation levels with Image Lab 3.0 software (Bio-Rad Laboratories) and normalized to appropriate loading controls.

### Statistical analyses

Statistical analyses were performed using either one-way ANOVA and Bonferonni's multiple comparison tests or Student's unpaired *t*-test with GraphPad Prism 6 (GraphPad Software; La Jolla, CA, USA). Data is presented as means ± SEM. Differences in results were considered statistically significant when *p* < 0.05.

## SUPPLEMENTARY MATERIALS FIGURES AND TABLES


