# Supplementary material for: β-cell insulin receptor deficiency during in utero development induces an islet compensatory overgrowth response
Source: Oncotarget. 2016 Jun 30;7(29):44927–40. doi: 10.18632/oncotarget.10342 (PMC5216695; doi:10.18632/oncotarget.10342)
Supplement: Supplementary file 1 [file oncotarget-07-44927-s001.pdf]

β-cell insulin receptor deficiency during *in utero* development induces an islet compensatory overgrowth response

Supplementary Material

Supplementary Table 1: List of Primers used for Polymerase Chain Reactions

Primer Name	Primer Pair Sequence
oIMR6765	5'- GAT GTG CAC CCC ATG TCT G - 3'
oIMR6766	5'- CTG AAT AGC TGA GAC CAC AG - 3'
MIP	5'- CCT GGC GAT CCC TGA ACA TGT CCT - 3'
CreER	5'- TGG ACT ATA AAG CTG GTG GGC AT - 3'

Supplementary Table 2: List of Antibodies used for Immunostaining and Western-Blot Analyses

Primary Antibodies		Dilution	Company
Anti-Akt	Rabbit polyclonal	1:3000*	Cell Signaling (Temecula, CA, USA)
Anti-Calnexin	Mouse monoclonal	1:1500*	BD Biosciences (Missasauga, ON, CA)
Anti-Caspase 3	Rabbit polyclonal	1:1000*	Cell Signaling (Temecula, CA, USA)
Anti-cleaved Caspase-3 (Asp175)	Rabbit polyclonal	1:200 / 1:1000*	Cell Signaling (Temecula, CA, USA)
Anti-GAPDH	Rabbit polyclonal	1:2000*	Santa Cruz Biotechnology (Santa Cruz, CA, USA)
Anti-Glucagon	Rabbit polyclonal	1:50	Santa Cruz Biotechnology (Santa Cruz, CA, USA)
Anti-Glut2	Rabbit polyclonal	1:100	Chemicon (Temecula, CA, USA)
Anti-Igf1	Rabbit polyclonal	1:200	Abcam (Cambridge, MA, USA)
Anti-Igf1rβ	Rabbit polyclonal	1:200	Cell Signaling (Temecula, CA, USA)
Anti-Igf2	Rabbit polyclonal	1:200 / 1:1000*	Abcam (Cambridge, MA, USA)
Anti-Insulin	Guinea pig polyclonal	1:50	Zymed (San Francisco, CA, USA)
Anti-Insulin	Mouse monoclonal	1:800	Sigma-Aldrich (St. Louis, MO, USA)
Anti-Insulin	Rabbit polyclonal	1:50	Santa Cruz Biotechnology (Santa Cruz, CA, USA)
Anti-Insulin Receptor	Mouse monoclonal	1:200 / 1:1000*	Millipore (Temecula, CA, USA)
Anti-Islet1	Mouse monoclonal	1:100	DSHB (University of Iowa, Iowa City, IA, USA)
Anti-Ki67	Rabbit polyclonal	1:100	Abcam (Cambridge, MA, USA)
Anti-MafA	Rabbit polyclonal	1:100	Bethyl Laboratory (Montgomery, TX, USA)
Anti-Nkx6.1	Mouse monoclonal	1:100	DSHB (University of Iowa, Iowa City, IA, USA)
Anti-p53	Mouse monoclonal	1:2000*	Cell Signaling (Temecula, CA, USA)
Anti-pan-Cytokeratin	Mouse monoclonal	1:50	Santa Cruz Biotechnology (Santa Cruz, CA,

			USA)
Anti-Pdx-1	Rabbit polyclonal	1:800	Dr. Wright (University of Vanderbilt, Nashville, TN, USA)
Anti-PECAM-1	Rabbit polyclonal	1:50	Santa Cruz Biotechnology (Santa Cruz, CA, USA)
Anti-phospho-Akt (Ser473)	Mouse monoclonal	1:2000*	Cell Signaling (Temecula, CA, USA)
Anti-phospho-p53 (Ser15)	Rabbit polyclonal	1:1000*	Cell Signaling (Temecula, CA, USA)
Anti-Vegf-a	Rabbit polyclonal	1:100 / 1:1000*	Abcam (Cambridge, MA, USA)
Anti-β-actin	Mouse monoclonal	1:5000*	Sigma-Aldrich (St. Louis, MO, USA)

* dilution factor applied to western blot analysis. DSHB, Developmental Studies Hybridoma Bank.JRL,

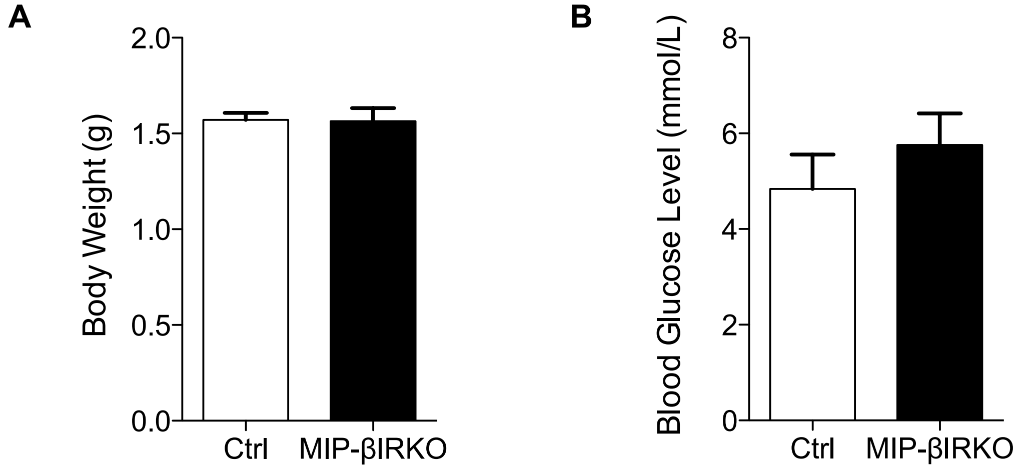

Supplementary Figure 1: Body weight and blood glucose levels in fetal MIP-βIRKO and control mice at e19-20. Body weight (A, n=16-18) and blood glucose levels (B, n=5-11) were measured at the time of fetal dissection. Quantification shows that fetal MIP-βIRKO mice displayed normal phenotypes comparable to control mice.

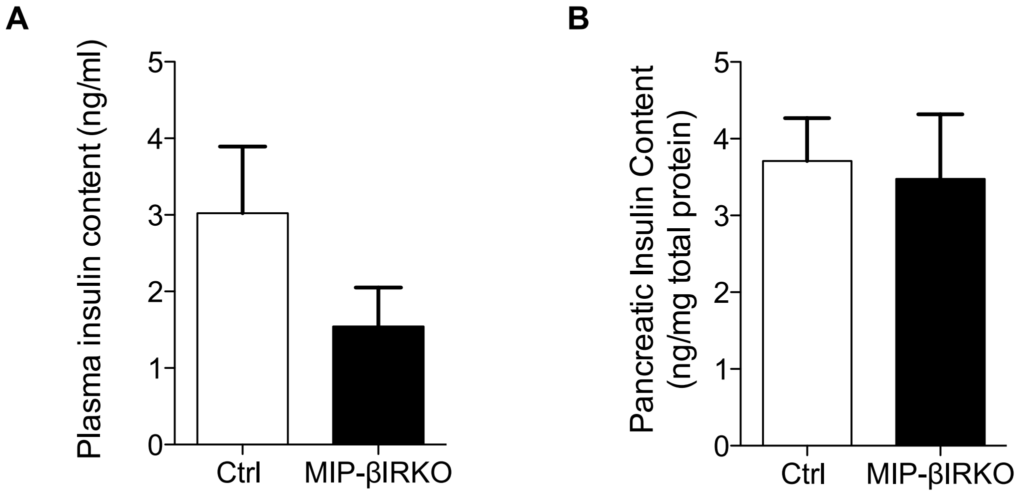

Supplementary Figure 2: Plasma insulin levels and pancreatic insulin content in fetal MIP-βIRKO and control mice at e19-20. Insulin levels were collected from plasma (A) and pancreatic (B) samples (n=3) at the time of fetal dissection. Similar circulating and pancreatic insulin content were found in fetal MIP-βIRKO mice compared to their control.

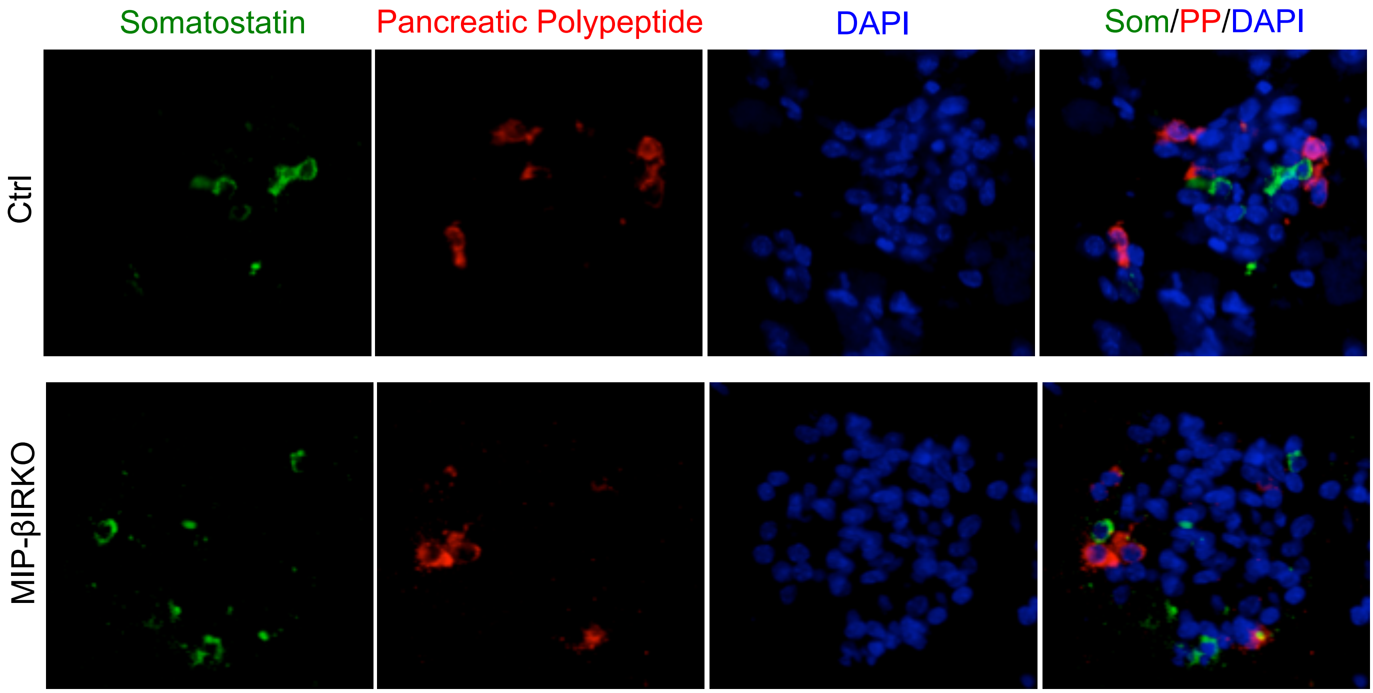

Supplementary Figure 3: Double immunostaining for somatostatin and pancreatic polypeptide in fetal MIP-βIRKO and control mice at e19-20.
